# Morphological and molecular study of Syllinae (Annelida, Syllidae) from Bermuda, with the description of five new species

**DOI:** 10.1098/rsos.230638

**Published:** 2023-08-23

**Authors:** Paula Moreno-Martín, Mar Mourín, Aida Verdes, Patricia Álvarez-Campos

**Affiliations:** ^1^ Centro de Investigación en Biodiversidad y Cambio global (CIBC-UAM), Departamento de Biología (Zoología), Facultad de Ciencias, Universidad Autónoma de Madrid, Madrid, Spain; ^2^ Departamento de Biodiversidad y Biología Evolutiva, Museo Nacional de Ciencias Naturales de Madrid, Consejo Superior de Investigaciones Científicas, MNCN-CSIC, Madrid, Spain

**Keywords:** *Haplosyllis*, *Opisthosyllis*, *Syllis*, systematics, diversity

## Abstract

Although polychaetes from the Bermuda Archipelago have been studied since the beginning of the twentieth century, syllids have been particularly neglected in this area, which is surprising considering this family is usually a dominant group in marine benthic ecosystems. To fill this knowledge gap, we have carried out an extensive analysis of Bermudan Syllidae, combining morphological and molecular data including four nuclear and mitochondrial markers (*cytochrome c oxidase subunit I*, *18S rRNA*, *16S rRNA* and *28S rRNA*). We have identified and established the phylogenetic position of five new species, *Haplosyllis anitae* n. sp., *Haplosyllis guillei* n. sp., *Haplosyllis larsi* n. sp., *Haplosyllis vassiae* n. sp. and *Syllis laiae* n. sp., together with *Haplosyllis* cf. *cephalata*. Overall, our results extend the knowledge on the diversity of Syllidae in Bermuda, increasing the number of species present in the area to 25. Our results also recover *Opisthosyllis* and *Syllis* as non-monophyletic genera, for which traditional diagnostic morphological features do not accurately reflect their evolutionary histories, and thus we propose that these groups should be reorganized based on molecular characters.

## 1. Introduction

**Figure 1.**
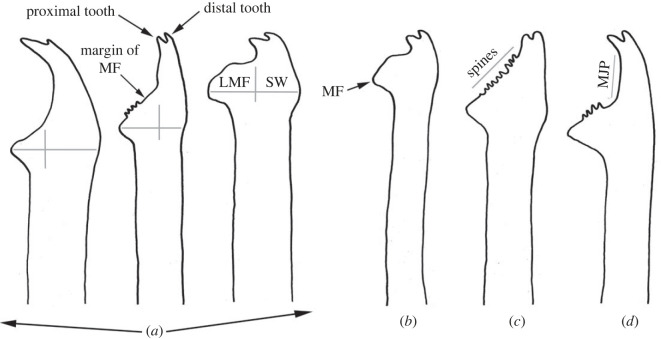
Most relevant features of simple chaetae in *Haplosyllis* species (adapted from Lattig & Martin [12]). (*a*) Comparison between length of main fang (LMF) and chaetal width (SW). (*b*–*d*) Different chaetal shapes according to mid-joining point (MJP). (*b*) Curved and short. (*c*) Diagonal and relatively long. (*d*) Straight and relatively long.

The Bermuda Archipelago comprises more than 150 islands and it is located in the West Atlantic Ocean. Throughout history, several oceanographic expeditions have been carried out in this area including the Challenger expedition in 1873, or the one led by Webster in 1884, who described and illustrated annelid species for the first time [1]. However, it was not until 1900, when Verrill made the first detailed descriptions of polychaete annelids, reporting 111 new species to science [2]. Since then, this number has increased and, currently, a total of 265 species belonging to 137 genera and 43 families of polychaete annelids have been documented in Bermuda [3]. Among them, the family Syllidae Grube, 1850 stands out for being one of the least studied groups in this area [4]. In fact, Verrill [2] was the first author who reported syllids from Bermuda, describing 24 new species, but some of them were later synonymized and currently, there are 19 valid species of Syllidae in the Bermuda Archipelago (electronic supplementary material, table S1). Syllids are very easy to recognize due to the presence of the proventricle, a muscularized region of the digestive tube, considered the autapomorphy of the family [5,6]. The family is currently divided into five subfamilies, of which Syllinae Grube, 1850 is the largest and most taxonomically complicated due to the lack of morphological synapomorphies for most of its genera [7]. Some of the most challenging genera within Syllinae include *Syllis*, that represents the type genus, and the closely related *Opisthosyllis*, both of which have been shown to be non-monophyletic in the most recent phylogenetic analyses [8–11]. *Syllis* and *Opisthosyllis* species have large bodies and conspicuous colorations, long and articulated appendages, and a huge variability in the morphology of their chaetae, a character that has been traditionally considered extremely important to differentiate species. In addition, all *Opisthosyllis* are characterized by a long pharynx with a tooth situated on its most posterior region, a feature that was considered the synapomorphy of the group but later shown to be phylogenetically uninformative [8]. Additionally, the current taxonomic organization of *Syllis* is not phylogenetically accurate since a clear synapomorphy has not been found for the entire group, and no robust solution has been proposed yet to resolve this issue [9]. On the contrary, the monophyly of another diverse genus within Syllinae, *Haplosyllis*, has not been questioned yet, but it also remains a poorly studied genus in several areas, especially due to the difficulties to properly recognize its species. The genus can be easily recognized by the presence of special simple chaetae (figure 1), a character considered the synapomorphy of the group, but the species are determined mainly by subtle details on these chaetae, and therefore, the correct differentiation among many species requires time and experience. In fact, until recently, most *Haplosyllis* specimens collected worldwide were assigned to *Haplosyllis spongicola*, a species living in symbiosis with different sponge species [13]. It was not until the shape of the chaetae and other morphological details were properly defined and considered (figure 1) that the huge diversity of the genus started to be recognized [14].

In Bermuda, only nine species within the Syllinae subfamily have been reported so far [2,4,15], including five species of *Syllis* (three spp.), *Opisthosyllis* (one sp.) and *Haplosyllis* (one sp.) (Verrill [2]; Hartman [15]; Pocklington [4]) (electronic supplementary material, table S1). Given the scarce number of studies and limited knowledge about the Syllinae subfamily in the Bermuda Archipelago, the aim of this work was to explore the diversity of syllines in the area, following an integrative taxonomical approach. We have collected more than 800 specimens from several points around the islands and performed an exhaustive morphological examination and a molecular multilocus analysis including two mitochondrial and two nuclear markers, to evaluate their taxonomic status and determine their phylogenetic position within the subfamily.

## 2. Material and methods

### 2.1. Sampling and morphological examination

A total of 803 specimens of Syllinae were collected during August of 2014 in Bermuda by snorkelling or SCUBA diving in different substrates and depths (electronic supplementary material, table S2). Specimens were sorted at the Bermuda Institute of Ocean Sciences (BIOS) using a Nikon SM7 stereoscope and preserved in 96% ethanol for the subsequent molecular and morphological analyses. Further examination and identification were completed at Universidad Autónoma de Madrid (UAM) using an Olympus SZ 40 stereoscope and an Olympus CX 43 light microscope. Drawings were made to scale with a camera lucida attached to a Nikon Optiphot light microscope and from pictures taken with an Olympus CX41 light microscope. The width of specimens was measured at the level of the proventricle, excluding the parapodia.

For scanning electron microscopy, selected specimens were prepared on an Emitech K850 Critical Point Dryer, gold-coated with a Q150T-S Turbo-Pumper Sputter Coater and examined with a Hitachi S 3000N scanning electron microscopy (SEM) at the Interdepartmental Research Service (SIDI) of the UAM.

Vouchers for all newly collected specimens were deposited at the Museo Nacional de Ciencias Naturales (MNCN) in Madrid (Spain). Catalogue numbers, collection dates, locality and additional relevant information are listed in electronic supplementary material, table S2.

### 2.2. DNA extraction, amplification and sequencing

Genomic DNA was extracted from two–three mid-body segments of 13 newly collected specimens, using the Speedtools Tissue Extraction Kit (Biotools) following the manufacturer's protocol. Fragments of the nuclear gene *18S rRNA* (approx. 1800 bp) and *28S rRNA* (approx. 530 bp) and the mitochondrial *16S rRNA* (approx. 470 bp) and *cytochrome c oxidase subunit I* (*COI*, approximately 650 bp) were polymerase chain reaction (PCR)-amplified. Three overlapping pairs of primers were used to amplify *18S rRNA*: 18S1F-18S5R, 18S4F-18S7R and 18Sa2.0–18S9R [16]. Primers 28Sa and 28Srd5b [17] were used to amplify *28S rRNA*. Primers 16SarL and 16SbrH [18] were used to amplify *16S rRNA* and primers jgLCO1490 and jgHCO2198 [19] were used to amplify *COI*.

Each PCR reaction consisted of 1.5 µl of DNA template in 13 µl reaction volumes containing 0.5 µl of each 10 mM primer and 12.5 µl of RED Taq DNA Polymerase (VWR, Avantor). The temperature profiles to amplify markers and to obtain the sequencing products are described in Álvarez-Campos *et al*. [10,20,21]. Using the forward primer of each pair described above, 10 µl of the PCR product were used for sequencing at the Servicio de Secuenciación Sanger, Unidad de Genómica, Universidad Complutense de Madrid (UCM).

Sequence data were edited in Geneious v. 6.1.6 [22]; primers were removed from all sequences and the three *18S rRNA* overlapping fragments were merged into a consensus sequence. The alignments for the three different genes were run in the online server of MAFFT v. 7 with default parameters [23].

### 2.3. Phylogenetic analyses

To evaluate the phylogenetic relationships between the newly collected Bermudan species and other Syllinae species, the 13 sequences generated here were combined with 48 sequences corresponding to 35 species available in GenBank (electronic supplementary material, table S2). A total of 42 species were incorporated in the analysis including representatives of *Haplosyllis* [10], *Opisthosyllis* [6], *Syllis* [10], *Branchiosyllis* [5], *Eurysyllis* Ehlers 1864 [3], *Plakosyllis* Hartmann-Schröder, 1956 [1] and *Trypanosyllis* [3]. Four species of *Perkinsyllis* San Martín, López & Aguado, 2009 were included as outgroups. All mitochondrial and nuclear datasets were analysed both individually and concatenated. Selection of models of sequence evolution was done using the Akaike information criterion (AIC) in JModeltest 2 [24]. The best model for the concatenated dataset was the general time reversible (GTR) with gamma-distributed rates across sites and a proportion of invariable sites (GTR + G + I).

The maximum-likelihood (ML) analysis of the concatenated partitioned dataset was run in RaxML v. 8.2.12 [25] using the GTR + G + I evolutionary model. Bootstrap support values were estimated using 1000 replicates and 10 starting trees [26]. Bayesian inference (BI) analyses were run in MrBayes v. 3.2.1 [27] using the GTR + G + I evolutionary model, with four Markov chains that were started from a random tree, running simultaneously for 20 million generations, with trees sampled every 200 generations (samplefreq = 200); the initial 25% of trees were discarded as burn-in (burninfrac = 0.25), after assessing for convergence with Tracer v. 1.6 [28]. The resulting phylogenetic trees were plotted in FigTree v.1.4.2 [28] and later edited with Illustrator CS5.

## 3. Results

### 3.1. Taxonomy

Family SYLLIDAE Grube, 1850

Genus ***Haplosyllis*** Langerhans, 1879

*Haplosyllis* Langerhans, 1879 [29, p. 527].

*Trypanosyllis* (*Trypanoseta*) Imajima & Hartman, 1964 [30, p. 129].

*Geminosyllis* Imajima, 1966 [31, p. 233]—Kudenov & Harris, 1995 [32, p. 71, fig. 1.26]—Lattig *et al*. 2007 [14, pp. 561–562].

*Trypanoseta* Aguado *et al*. 2008 [33, p. 544].

**Type species.** *Haplosyllis spongicola* (Grube, 1855)

**Diagnosis**. Cylindrical body, medium to large in size. Rounded prostomium with four eyes and sometimes with two anterior eyespots. Palps robust, fused at the base. Anterior appendages with specific alternation pattern for each species, with dorsal cirri long (L), medium (M) or short (S) in the first five segments. Parapodia only with simple chaetae, very variable in number, normally from one to three chaetae, but up to 12 in some species. Reproduction typically by acephalous stolons with no anterior appendages [5].

**Remarks**. Several specific parts of the chaetae have been defined to correctly describe and identify *Haplosyllis* species [12,13]: length of main fang (LMF), chaetal width (SW), upper side of the main fang (MF), mid-joining point (MJP) (figure 1).

*Haplosyllis* cf. *cephalata* **Verrill, 1900**

Figures 2 and 3

**Figure 2.**
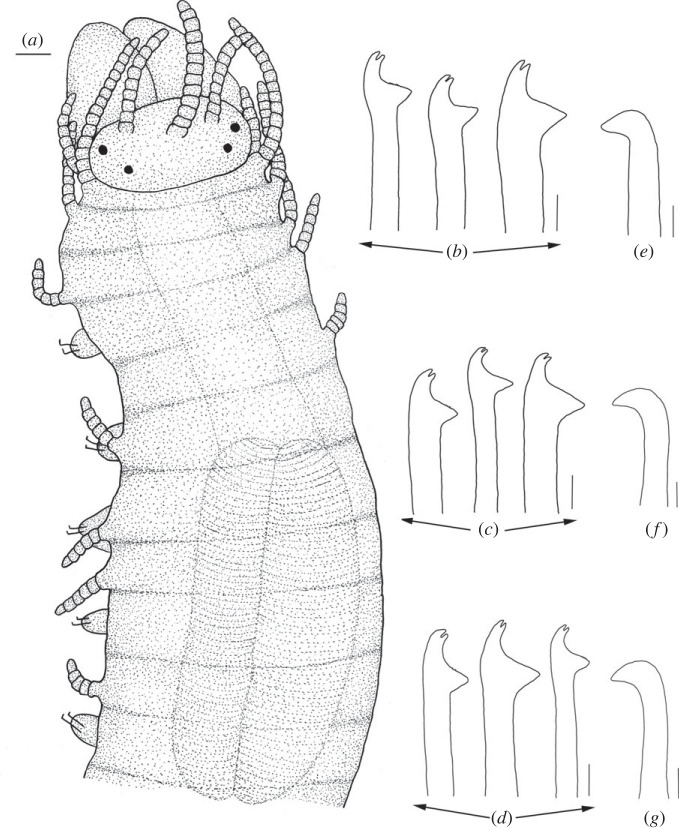
*Haplosyllis* cf. *cephalata* line drawing (MNCN 16.01/19292). (*a*) Anterior part, dorsal view. (*b*) Simple chaetae, anterior parapodia. (*c*) Simple chaetae, mid-body parapodia. (*d*) Simple chaetae, posterior parapodia. (*e*) Acicula, anterior parapodia. (*f*) Acicula, mid-body parapodia. (*g*) Acicula, posterior parapodia. Scale bars: (*a*) 0.195 mm; (*b*–*g*) 5 µm.

**Figure 3.**
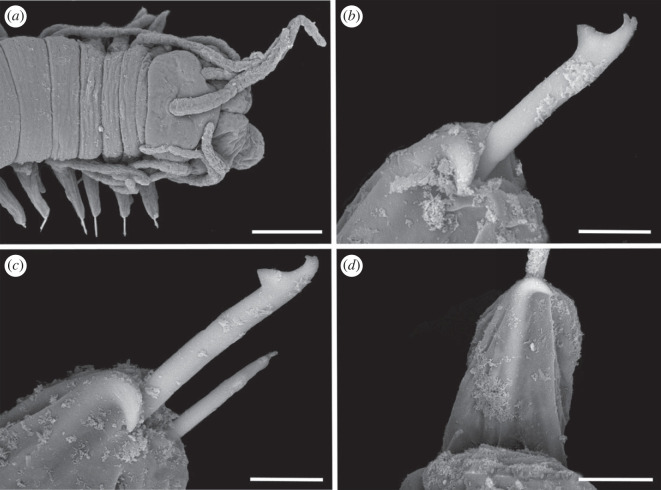
Scanning electron micrographs of *Haplosyllis* cf. *cephalata* (MNCN 16.01/19293). (*a*) Anterior part, dorsal view. (*b*) Simple chaeta, anterior parapodia. (*c*) Simple chaetae, posterior parapodia. (*d*) Acicula, posterior parapodia. Scale bars: (*a*) 100 µm; (*b–d*) 10 µm.

*Haplosyllis cephalata* Verrill, 1900 [2, pp. 613–614]—Licher, 1999 [34, p. 280].

**Material examined**. Ninety-five specimens fixed in 96% EtOH (MNCN 16.01/19292), one mounted for SEM (MNCN 16.01/19293) and mid-body segments of two specimens used for molecular analyses (MNCN 16.01/19294; MNCN 16.01/19295 and MNCN ADN 150066). Bermuda, Whalebone Bay, St George's Island (32°21'53.5” N, 64°42'45.4” W), inside an unidentified red sponge, 2 m depth, 8 August 2014.

**Comparative material examined.** *Haplosyllis cephalata*, syntypes, seven specimens mounted in a microscope slide, Yale Peabody Museum (YPM IZ 022544) Bermuda, Atlantic Ocean, A.E. Verrill Bermuda Expedition.

**Diagnosis**. Robust bodies with large palps (longer than prostomium) and anterior cirri with specific alternation pattern (L-M-S-S-S-S): dorsal tentacular cirri long with 14–16 articles; cirri of first chaetiger slightly shorter, with approximately 11–12 articles; cirri of second to fifth chaetigers decreasing in length with approximately 5–9 articles. All mid-body and posterior cirri with 5 articles. One or two chaetae per parapodium with a slightly curved and long MJP, with very small spines on the upper side of the MF. One distally bent acicula protruding from each parapodia, thicker in posterior ones.

**Description**. Complete specimen, 5 mm long, 0.6 mm wide, 35 segments. No coloration observed. Prostomium with two pairs of reddish-brown eyes in trapezoidal arrangement, anterior ones slightly larger than posterior ones (figure 2*a*). Palps longer than prostomium, fused only at base. Central antenna inserted in middle of prostomium, longer than lateral ones, with 13–14 articles. Lateral antennae inserted on anterior margin of prostomium, each with 9–10 articles (figures 2*a* and 3*a*). Dorsal tentacular cirri similar to lateral antennae, with 14–16 articles. Ventral tentacular cirri with 6–7 articles. Dorsal cirri of first chaetiger with 12 articles; cirri of second to fifth chaetigers with 5–9 articles; rest of cirri decreasing in length, with approximately 5 articles (figures 2*a* and 3*a*). Ventral cirri digitiform, similar to or shorter than parapodial lobes. One or two simple bidentate chaetae per parapodium, with proximal tooth slightly larger than distally one (figure 2*a*). Some chaetae with both teeth directed forwards, some directed upward. MJP curved, slightly shorter in anterior chaetae (figures 2*b–d* and 3*b,c*). MF pointed, with upper side straight in anterior chaetae and curved directed downwards in mid-body and posterior chaetae. Small spines on upper side of MF, only visible under the microscope (figures 2*b–d* and 3*b, c*). LMF similar in all chaetae, slightly shorter or equal to SW (figures 2*b–d* and 3*b,c*). One large and distally bent acicula per parapodium, some of them protruding from it (figures 2*e–g* and 3*b–d*). Pharynx extending through 5–6 segments, tooth on anterior margin. Large barrel-shaped proventricle extending through 5 segments, with approximately 38–41 muscle cell-rows (figure 2*a*). Anal cirri with 6–10 articles.

**Remarks**. The YPM specimens collected by Verrill [2] in Bermuda agree with our specimens in the general morphology of the body and the length of dorsal cirri. However, most of the chaetae in the syntypes are lost or broken and, thus, specific details such as the length of teeth or the spines on margin of the main fang cannot be properly observed. In addition, several parapodia present the same large and distally bent acicula, but in some anterior parapodia there are individuals showing two straight aciculae, which were not visible in any of our specimens. *Haplosyllis cephalata* was also reported in Colombia [12,35], but the specimens present dorsal papillae [35] that were not observed in specimens from the type locality (figures 2 and 3) [2]. In addition, the MJP of anterior and mid-body chaetae in the Colombian specimens are less curved, and the spines of the MF shorter than in Bermudan individuals [35]. Given these morphological differences it is possible that the Colombian and Bermudan specimens represent distinct species, but this cannot be appropriately evaluated until fresh material from Colombia is available for molecular analyses. In addition, the Caribbean species *Haplosyllis gula* Treadwell, 1924, presents some similarities with *Haplosyllis* cf. *cephalata* in the length and size of the proximal tooth, especially on the anterior chaetae, since they are both slightly upwards directed and moderately larger than the distal one. However, both species differ in the length of cirri along the body, since in *H. gula*, cirri length alternates from the sixth chaetiger while in *Haplosyllis* cf. *cephalata* cirri length decreases from the fifth chaetiger. Furthermore, the morphology of posterior chaetae is quite different, with *H. gula* having narrower chaetae with longer MJP not seen in *Haplosyllis* cf. *cephalata* [35].

**Habitat**. Symbiont of unidentified sponges.

**Distribution**. North-Western Atlantic Ocean and Caribbean Sea.

*Haplosyllis anitae* n. sp.

Figures 4 and 5.

**Figure 4.**
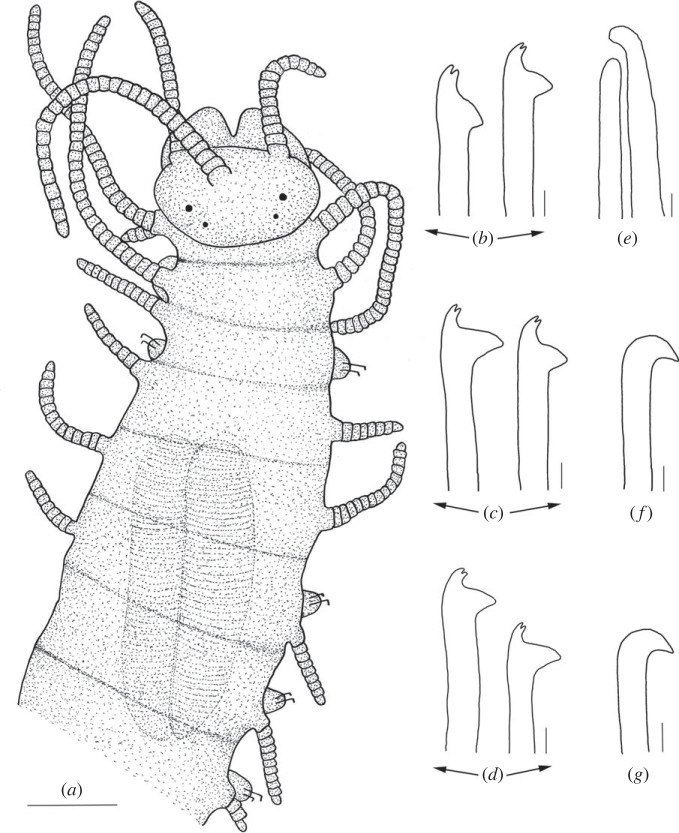
*Haplosyllis anitae* n. sp. line drawing (MNCN 16.01/19296). (*a*) Anterior part, dorsal view. (*b*) Simple chaetae, anterior parapodia. (*c*) Simple chaetae, mid-body parapodia. (*d*) Simple chaetae, posterior parapodia. (*e*) Aciculae, anterior parapodia. (*f*) Acicula, mid-body parapodia. (*g*) Acicula, posterior parapodia. Scale bars: (*a*) 0.195 mm; (*b–g*) 5 µm.

**Figure 5.**
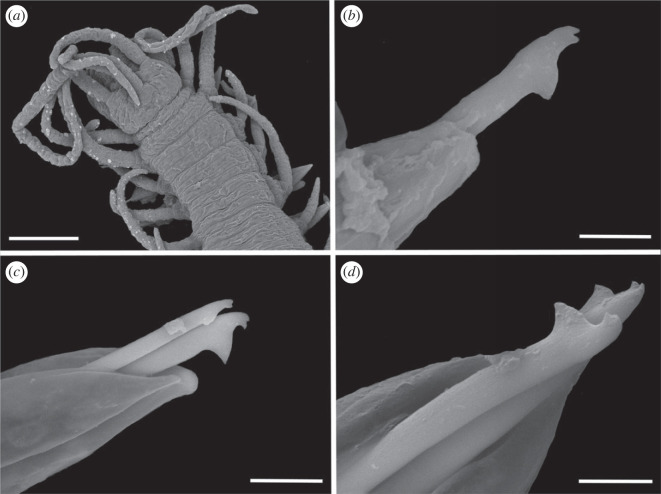
Scanning electron micrographs of *Haplosyllis anitae* n. sp. (MNCN 16.01/19298). (*a*) Anterior part, dorsal view. (*b*) Simple chaeta, anterior parapodia. (*c*) Simple chaetae, mid-body parapodia. (*d*) Simple chaetae, mid-body parapodia. Scale bars: (*a*) 100 µm; (*b,d*) 5 µm; (*c*) 10 µm.

Zoobank ID: urn:lsid:zoobank.org:act:34A34EA8-60C3-4CEF-BCE8-525F7F330B44

**Material examined**. Holotype (MNCN 16.01/19296), 361 paratypes fixed in 96% EtOH (MNCN 16.01/19297), one mounted for SEM (MNCN 16.01/19298) and mid-body segments of two specimens used for molecular analyses (MNCN 16.01/19299/MNCN 16.01/19300; MNCN ADN 150067 and 150068, respectively). Bermuda, Whalebone Bay, St George's Island (32°21′53.5″ N, 64°42′45.4″ W), inside unidentified red sponge, 2 m depth, 8 August 2014.

**Comparative material**. *Haplosyllis eldagainoae* Lattig & Martin, 2011. One paratype (MNCN 16.01/13170) Tanzania, Zanzíbar Canal, Bagamoyo, Mwanda, 6°22′40″ S, 38°58′40″ E, in small coral reef, inside the sponge *Theonella swinhoei*, 15–20 m depth, August 1996; one specimen (MNCN16.01/1317) in a fringing reef near Hurghada (Gulf of Suez), Red Sea, Egypt, 27°35′36.60″ N, 33**°**53′3.12″ E, inside the sponge *Theonella swinhoei*, 7–22 m depth.

**Diagnosis**. Small body and anterior cirri with specific alternation pattern (L-L-M-S-M-S): dorsal tentacular cirri and cirri of first chaetiger considerably longer than the rest (29–33 articles), cirri of second and fourth chaetiger much shorter (12–15 articles), cirri of third and fifth chaetiger slightly decreasing in length [8–10] until the posterior ones (5–7 articles). MJP short and curved, especially in posterior chaetae, with MF without spines and progressively longer towards the posterior part of the body.

**Description**. Holotype: complete specimen with small body size, 2 mm long, 0.4 mm wide, 24 segments. Prostomium rounded with two pairs of reddish-brown eyes in trapezoidal arrangement, anterior ones larger than posterior ones (figure 4*a*). Palps slightly shorter than prostomium, fused only at the base. Central antenna inserted in the middle of prostomium, longer than lateral antennae with 30–31 articles (figures 4*a* and 5). Lateral antennae inserted on anterior margin of prostomium, each with 15–16 articles. Dorsal tentacular cirri similar to central antennae, with 29–30 articles. Ventral tentacular cirri with 5–6 articles (figure 4*a*). Dorsal cirri of first chaetiger similar in length to dorsal tentacular cirri, with 32–33 articles; cirri of second and fourth chaetiger considerably shorter with 12–13 and 14–15, respectively; cirri of third and fifth chaetiger with 8–10 articles; rest of cirri decreasing in length, posterior cirri with 5–7 articles (figures 4*a* and 5*a*). Ventral cirri digitiform, similar to or shorter than parapodial lobes. One or two chaetae per parapodium, bidentate with the two teeth similar in size. MJP curved, relatively longer in anterior chaetae than in mid-body and posterior ones (figures 4*b–d* and 5*b–d*). MF smaller in anterior chaetae, increasing in posterior ones with the upper side of the MF straighter and forward directed in posterior chaetae (figures 4*b–d* and 5*b–d*). No spines visible on upper side of MF (figures 4*b–d* and 5*b–d*). When two chaetae are present, SW of both chaetae are similar to LMF (figures 4*b–d* and 5*c*,*d*). LMF increases progressively from anterior to posterior chaetigers, anterior chaetae with LMF equal to SW and posterior chaetae with LMF longer than SW (figures 4*b–d* and 5*b–d*). Broad aciculae, some protruding from parapodia. Two aciculae in anterior parapodia, both with curved end (figure 4*e*). One single and thick, curved and distally pointed acicula in mid-body and posterior parapodia (figure 4*f–g*). Pharynx short, extending approximately 3 segments, tooth on the anterior margin. Small barrel-shaped proventricle, extending approximately 3–4 segments with approximately 38–40 muscle cell-rows (figure 4*a*). Anal cirri short, with 3–4 articles.

**Remarks***. Haplosyllis anitae* n. sp. is similar to *Haplosyllis eldagainoae* Lattig & Martin, 2011 from the Indian Ocean in the morphology of mid-body and posterior aciculae, as well as in the chaetae with short and curved MJP in both species. However, the new species is larger and more robust than the Indian one, and they differ on the shape of chaetae, since the MF is shorter and more curved *H. eldagainoae* than in *H. anitae* n. sp. [36]. The studied paratype of *H. eldagainoae* (16.01/13170) is similar to *H. anitae* n. sp. in the general shape of both teeth and the MJP of the chaetae, being short, as well as the general shape of mid-body and posterior aciculae. However, they greatly differ in the MF, which is shorter and more curved in *H. eldagainoae*, while the body length is larger in the Bermudan species. On the other hand, the specimen of *H. eldagainoae* studied from the Red Sea (16.01/13171) shares some similarities with the new species in the shape of the chaetae, with curved MJP and short MF on anterior chaetae, increasing in posterior ones. However, it differs from the new species in the general morphology of the body and in the posterior aciculae which have less curved and less pointed tips than *H. anitae* n. sp., whose aciculae have robust, curved and distally pointed tips.

**Habitat**. Symbiont of unidentified red sponge, 2 m depth.

**Distribution**. West Atlantic Ocean, Bermuda Archipelago.

**Etymology**. Named after spongologist Dr. Ana Riesgo (MNCN), Anita, colleague and friend of A.V. and P.Á.-C. for her great help, generosity and mentorship throughout the years.

*Haplosyllis guillei* n. sp.

Figures 6 and 7

**Figure 6.**
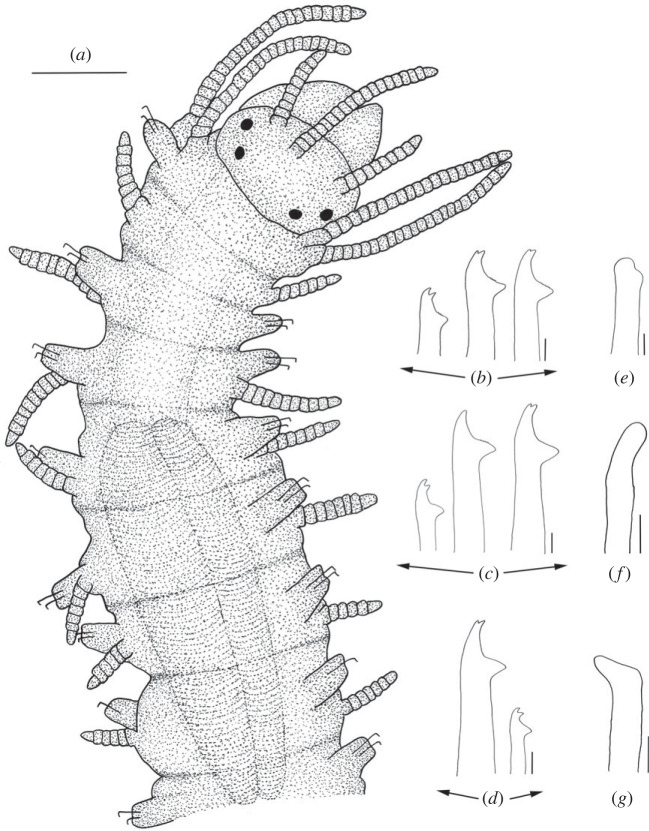
*Haplosyllis guillei* n. sp. line drawing (MNCN 16.01/19301). (*a*) Anterior part, dorsal view. (*b*) Simple chaetae, anterior parapodia. (*c*) Simple chaetae, mid-body parapodia. (*d*) Simple chaetae, posterior parapodia. (*e*) Acicula, anterior parapodia. (*f*) Acicula, mid-body parapodia. (*g*) Acicula, posterior parapodia. Scale bars: (*a*) 0.195 mm; (*b–g*) 5 µm.

**Figure 7.**
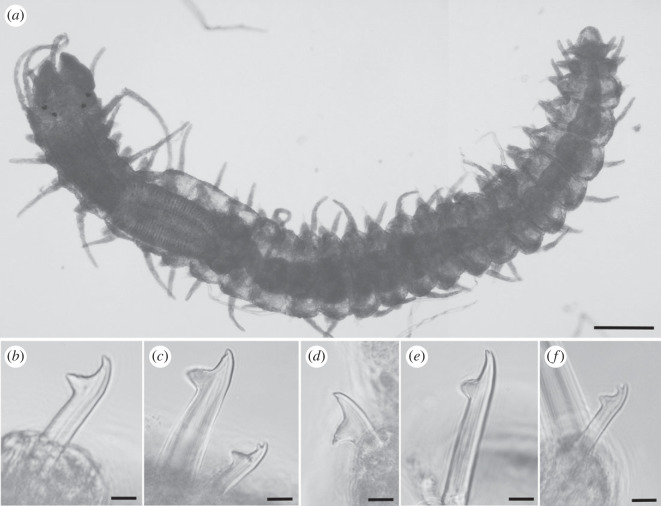
Light microscopy images of *Haplosyllis guillei* n. sp. (MNCN 16.01/19301). (*a*) Complete specimen dorsal view. (*b*) Simple chaeta, anterior parapodia. (*c*) Simple chaetae, anterior–mid-body parapodia. (*d*) Simple chaeta, mid-body parapodia. (*e*) Simple chaetae, mid-body–posterior parapodia. (*f*) Simple chaeta, posterior parapodia. Scale bars: (*a*) 200 µm; (*b–f*) 5 µm.

Zoobank ID: urn:lsid:zoobank.org:act:D7E908A5-8B41-469B-A1BB-B0CA510E4C5D

**Material examined**. Holotype (MNCN 16.01/19301), one paratype fixed in 96% EtOH (MNCN 16.01/19302). Bermuda: three specimens in 96% EtOH and mid-body segments of one specimen used for molecular analyses (MNCN 16.01/19303; MNCN ADN 150069), Bermuda, Ferry Reach, St George's Island (32°22′42″ N, 64°40′49″ W), unidentified red sponge, 3 m depth, 10 August 2014.

**Diagnosis**. Medium-sized body with specific alternation pattern in anterior cirri (L-L-S-S-S-S). Dorsal tentacular cirri and cirri of first chaetiger similar in length to cirri of first chaetiger, with 19–20 and 22–23 articles respectively. Cirri decreasing in length from second chaetiger towards posterior end, with approximately 7–10 chaetigers. Usually, two bidentate chaetae per parapodia, with proximal tooth slightly larger than distal one in anterior chaetae, and MF with a few small spines. Some mid-body chaetae unidentate.

**Description**. Holotype: complete specimen, medium size and slender, 2.5 mm long, 0.3 mm wide, with 27 segments. Prostomium rounded with two pairs of reddish-brown eyes in trapezoidal arrangement, similar in size (figures 6*a* and 7*a*). Palps of medium size, similar in length to prostomium, slightly longer and fused in the basal area (figures 6*a* and 7*a*). Central antenna inserted in the middle of the prostomium, slightly longer than lateral ones; central one with approximately 19–20 articles, lateral ones with 9–10 articles (figures 6*a* and 7*a*). Dorsal tentacular cirri with approximately 19–20 articles and ventral tentacular cirri with 7–9 articles (figures 6*a* and 7*a*). Cirri of first chaetiger slightly longer than dorsal tentacular cirri, with approximately 22–23 articles. From second to fifth chaetigers, cirri with approximately 6–10 articles. Mid-body and posterior cirri reduced to 6–8 articles (figures 6*a* and 7*a*). Ventral cirri digitiform, similar to or shorter than parapodial lobes. Usually, two bidentate chaetae on each parapodium (figure 6*a*). Anterior chaetae with proximal tooth slightly larger than distal one, short and curved MJP, with few small spines on the upper side of the MF, LMF smaller than SW (figures 6*b* and 7*b*,*c*). Mid-body chaetae with proximal and distal tooth very reduced, becoming in some cases unidentate chaetae (figures 6*c* and 7*c*); MJP more curved and slightly longer than anterior ones; small spines in the upper side of the MF in some chaetae (figures 6*c* and 7*c–e);* LMF smaller than SW. Posterior chaetae similar to anterior ones, with two chaetae. Large chaetae with proximal tooth larger than distal one, MJP straight, LMF twice smaller than SW and with few small spines in the upper side of the MF (figures 6*d* and 7*e*). Small chaetae with both teeth similar in size, diagonal MJP, LMF larger than SW and few small spines in the upper side of the MF (figures 6*d* and 7*f*). One or two aciculae in anterior parapodia, distally blunt; one acicula in mid-body and posterior parapodia, distally curved and slightly rounded (figure 6*e–g*). Pharynx extending through 4–5 segments, tooth on anterior margin. Barrel-shaped proventricle similar in length to pharynx, extending through 6–7 segments, with 30–32 muscle cell-rows (figures 6*a* and 7*a*). Anal cirri with 10–11 articles.

**Remarks**. The morphology of the chaetae in *Haplosyllis guillei* n. sp. resembles that of *Haplosyllis uncinigera* (Grube, 1878), being bidentate with a very small distal tooth in some mid-body and posterior chaetae, sometimes giving a unidentate appearance with small spines in the upper side of the MF of anterior and mid-body chaetae. In addition, both species have two chaetae of different sizes, with a small chaeta being clearly bidentate. However, they differ in the number and morphology of the aciculae, with *H. uncinigera* bearing 4–5 aciculae with curved pointed tips in anterior parapodia, and 3–4 aciculae in mid-body and posterior parapodia with pointed, straight or curved tips; in contrast, *H. guillei* n. sp. only has 1–2 distally blunt aciculae in anterior parapodia and one acicula in mid-body and posterior parapodia with slightly rounded tip [29].

**Habitat**. Symbiont of unidentified red sponge, 3 m depth.

**Distribution**. West Atlantic Ocean, Bermuda Archipelago.

**Etymology**. The species is named in honour of Prof. Guillermo San Martín (UAM), world-renowned syllid expert, for his contributions to the field of polychaete research, and for always sharing his experience and knowledge with the authors.

*Haplosyllis larsi* n. sp.

Figures 8 and 9

**Figure 8.**
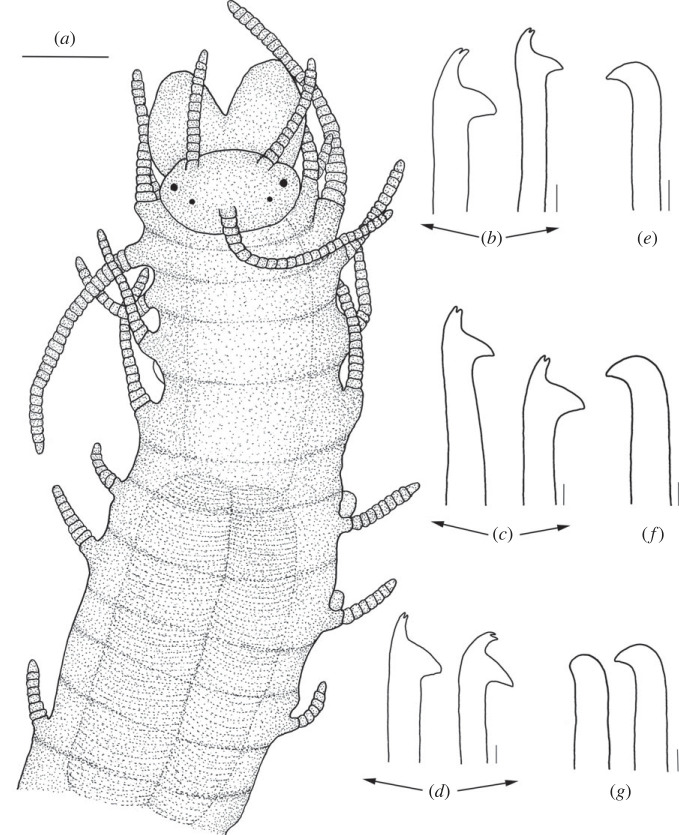
*Haplosyllis larsi* n. sp. line drawing (MNCN 16.01/19304). (*a*) Anterior part, dorsal view. (*b*) Simple chaetae, anterior parapodia. (*c*) Simple chaetae, mid-body parapodia. (*d*) Simple chaetae, posterior parapodia. (*e*) Acicula, anterior parapodia. (*f*) Acicula, mid-body parapodia. (*g*) Aciculae, posterior parapodia. Scale bars: (*a*) 0.375 mm; (*b–g*) 5 µm.

**Figure 9.**
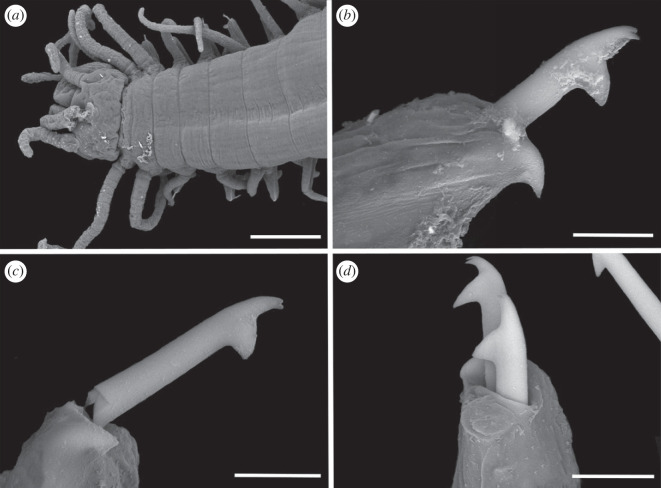
Scanning electron micrographs of *Haplosyllis larsi* n. sp. (MNCN 16.01/19306). (*a*) Anterior part, dorsal view. (*b*) Simple chaetae and acicula, anterior parapodia. (*c*) Simple chaeta and acicula, mid-body parapodia. (*d*) Simple chaetae, posterior parapodia. Scale bars: (*a*) 100 µm; (*b–g*) 10 µm.

Zoobank ID: urn:lsid:zoobank.org:act:E7C2D753-E388-4562-BD1C-0824871305A9

**Material examined**. Holotype (MNCN 16.01/19304), 229 paratypes fixed in 96% EtOH (MNCN 16.01/19305), one mounted for SEM (MNCN 16.01/19306) and mid-body segments of two specimens used for molecular analyses (MNCN 16.01/19307; MNCN 16.01/19308; MNCN ADN 150070). Bermuda, BIOS dock, St George's Island (32°22′13.9″ N, 64°41′47.7″ W), inside unidentified blue sponge, 2 m depth, 13 August 2014.

**Diagnosis**. Medium-sized body and anterior cirri with specific alternation pattern (S-L-S-S-M-S): dorsal tentacular cirri shorter that cirri in first chaetiger with 14–16 and 26–28 articles respectively; cirri of second, third and fifth cirri similar in length to tentacular cirri with 12–14 articles, and cirri of fourth chaetiger slightly larger with 18–20 articles. Chaetae with MJP longer in anterior parapodia than in mid-body and posterior ones, without spines on the upper side of the MF, which is curved. Posterior chaetae with proximal tooth slightly longer than distal one. One single robust, curved and pointed acicula on each parapodium.

**Description**. Holotype: complete specimen, medium size, 3 mm long, 0.6 mm wide, 29 segments. Prostomium rounded with two pairs of reddish-brown eyes in trapezoidal arrangement, anterior ones larger than posterior ones (figures 8*a* and 9*a*). Palps slightly longer than prostomium, fused only at base. Central antenna inserted in the middle of prostomium, longer than lateral ones, with 23–25 articles. Lateral antennae inserted in the anterior margin of prostomium, with 12–14 articles. Dorsal tentacular cirri similar in length to lateral antennae, with 14–16 articles (figures 8*a* and 9*a*). Ventral tentacular cirri with 5 articles. Dorsal cirri of first chaetiger much longer, with 26–28 articles; cirri of second, third and fifth chaetigers similar in length to dorsal tentacular cirri, with 12–14 articles; cirri of fourth chaetiger with 18–20 articles. Mid-body cirri decrease in length to 7–9 articles, and posterior ones to 3–6 articles (figure 8*a*). Ventral cirri digitiform, similar to, or shorter than parapodial lobes. One or two chaetae per parapodium, bidentate with proximal tooth slightly longer than distal tooth in some chaetae (figures 8*b–d* and 9*b–d*). Both teeth moderately forward directed, particularly in some posterior chaetae. MF pointed, rather large in posterior chaetae, with upper side slightly downward directed in all chaetae, especially on posterior ones (figures 8*b–d* and 9*b–d*). No spines on the upper side of MF. Long and curved MJP in anterior chaetae, straighter and shorter in mid-body and posterior ones (figures 8*b–d* and 9*b–d).* LMF shorter than SW in anterior chaetae, and equal in length in mid-body and posterior ones (figures 8*b–d* and 9*b–d*). One robust, curved and pointed acicula in anterior and mid-body parapodia, two aciculae in posterior ones, larger one similar to anterior ones, smaller acicula thinner, with rounded tip (figures 8*e–f* and 9*b*,*c*). Pharynx extending approximately 6 segments, with tooth on anterior margin. Large, barrel-shaped proventricle, extending approximately 6 segments, with 33–36 muscular cell-rows (figure 8*a*). Anal cirri with 6–8 articles.

**Remarks**. *Haplosyllis larsi* n. sp. is similar to the Caribbean species *H. chaetafusorata* and *H. niphatesicola* in the morphology of the chaetae [34]. *Haplosyllis chaetafusorata* and *H. larsi* n. sp. have straight MJP, the LMF similar to SW and the proximal tooth slightly longer than the distal one, but they differ in the presence of granules in the dorsal surface of *H. chaetafusorata* together with the spines present on the upper side of the MF, not present in the new species. *Haplosyllis niphatesicola* has posterior chaetae with a straight MJP and a long and pointed MF, similar to *H. larsi* n. sp. but it also presents small teeth on the upper side of the MF unlike the new species [35]. The Indo-Pacific species *Haplosyllis leylae* Cepeda, Martin, Britayyev, Al Aidaroos & Lattig, 2017 also shares some similarities in the shape of chaetae with *H. larsi* n. sp., with the proximal tooth longer than the distal one in some chaetae, and the MJP straight and long in posterior chaetae, with LMF similar in length to SW. They also have the same number and the general shape of aciculae, with 1–2 on each parapodia, either with straight tips or with long, pointed and curved tips. However, both species greatly differ in the length of cirri, which are generally longer along the body in *H. larsi* n. sp. while decreasing in length in *H. leylae* along the body, until chaetiger 8 onwards where cirri have only one article [37].

**Habitat**. Symbiont of unidentified blue sponge, 2 m depth.

**Distribution**. West Atlantic Ocean, Bermuda Archipelago.

**Etymology**. Named after spongologist Dr Lars Kumala (University of Southern Denmark), colleague and friend of A.V. and P.Á.-C. for his help and wonderful companionship during the authors' stay at the Bermuda Institute of Ocean Sciences.

*Haplosyllis vassiae* n. sp.

Figures 10 and 11

**Figure 10.**
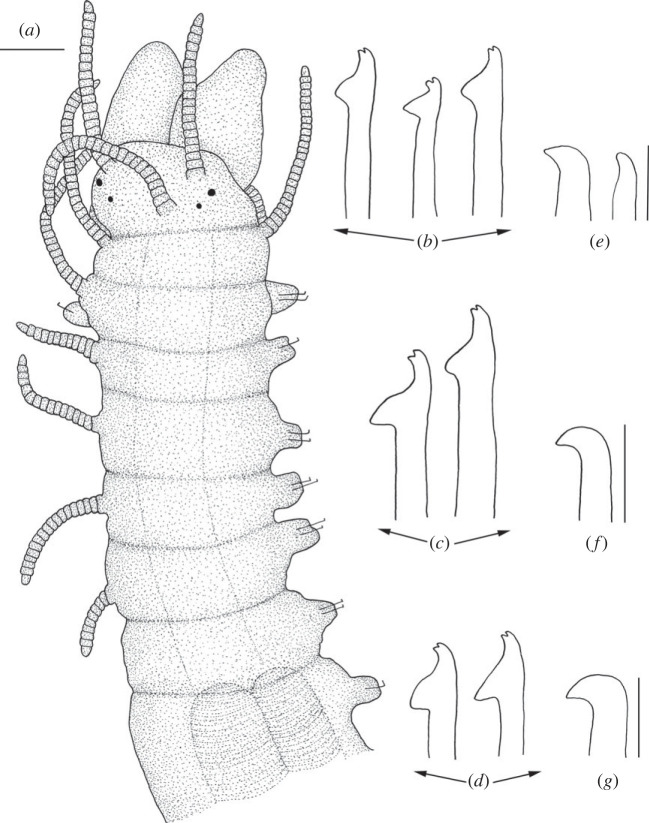
*Haplosyllis vassiae* n. sp. line drawing (MNCN 16.01/19309). (*a*) Anterior part, dorsal view. (*b*) Simple chaetae, anterior parapodia. (*c*) Simple chaetae, mid-body parapodia. (*d*) Simple chaetae, posterior parapodia. (*e*) Aciculae, anterior parapodia. (*f*) Acicula, mid-body parapodia. (*g*) Acicula, posterior parapodia. Scale bars: (*a*) 0.195 mm; (*b–g*) 5 µm.

**Figure 11.**
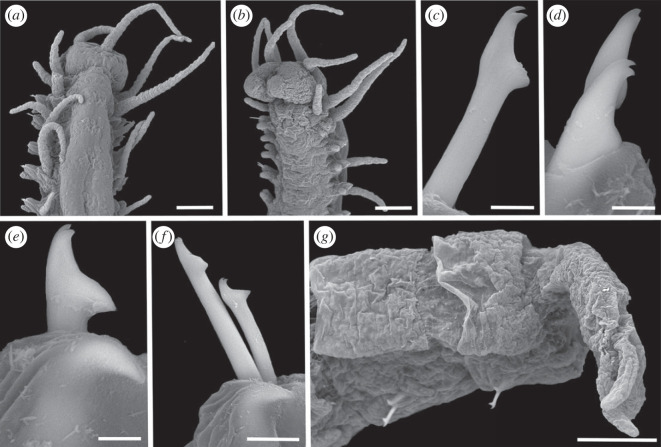
Scanning electron micrographs of *Haplosyllis vassiae* n. sp. (MNCN 16.01/19311). (*a*) Anterior part, dorsal view. (*b*) Anterior part, dorsal view. (*c*) Simple chaeta, anterior parapodia. (*d*) Simple chaetae, anterior–mid-body parapodia. (*e*) Simple chaeta and acicula, mid-body parapodia. (*f*) Simple chaetae and acicula, midbody-posterior parapodia. (*g*) Pygidium. Scale bars: (*a*,*b*) 100 µm; (*c–e*) 5 µm; (*f*) 10 µm; (*g*) 100 µm.

Zoobank ID: urn:lsid:zoobank.org:act:A036DDE1-1156-45BE-94D0-096A45CF72B2

**Material examined**. Holotype (MNCN 16.01/19309), 97 paratypes fixed in 96% EtOH (MNCN 16.01/19310), one mounted for SEM (MNCN 16.01/19311) and mid-body segments of two specimens used for molecular analyses (MNCN 16.01/19312; MNCN ADN 150071). Bermuda, Ferry Reach, St George's Island (32°22′42″ N, 64°40′49″ W), inside unidentified yellow sponge, 2m depth; 13 August 2014.

**Diagnosis**. Large body with large palps almost twice as long as prostomium. Anterior cirri with specific alternation pattern (L-L-S-S-S-S): dorsal tentacular cirri and cirri of first chaetiger with 25–26 articles; second to fifth cirri with 10–12 articles. Bidentate chaetae, without spines in the upper side of the MF. LMF shorter than MJP in anterior chaetae and larger in posterior ones.

**Description**. Holotype: complete specimen, large and slender body, 5.2 mm long, 0.5 mm wide, 30 segments. Prostomium rounded with two pairs of reddish eyes in trapezoidal arrangement, anterior eyes slightly larger than posterior ones (figures 10*a* and 11*a,b*). Large palps, almost twice the length of the prostomium, fused in the most basal area (figures 10*a* and 11*a,b*). Central antenna inserted in the middle of prostomium, slightly longer than lateral ones. Central antenna with 25–26 articles and lateral antennae with 19–20 articles (figures 10*a* and 11*a,b*). Dorsal tentacular cirri with approximately 24–26 articles, ventral ones with 9–10 articles (figures 10*a* and 11*a,b).* Cirri of first chaetiger similar in length to dorsal tentacular cirri, with 24–26 articles. Cirri of second to fifth chaetigers with approximately 10–12 articles. Mid-body and posterior cirri with approximately 4–5 articles. Ventral cirri digitiform, smaller than parapodial lobes. One or two chaetae on each parapodium, bidentate, with both teeth similar in size and in most cases without spines on the upper side of the MF (figure 10*a–d*). Straight and long MJP in anterior chaetae with pointed MF and in some cases with small spines on its upper side (figure 11*c*). LMF similar or smaller than SW in anterior chaetae (figures 10*b* and 11*c*,*d*). MJP more curved and shorter in mid-body and posterior chaetae. LMF very similar to SW in mid-body and posterior chaetae (figures 10*c,d* and 11*c–f).* One or two aciculae in anterior parapodia, one relatively thick, with curved and pointed tip, the other one thinner with less curved and pointed tip (figure 10*e*). One robust, curved and pointed acicula in mid-body and posterior parapodia (figures 10*f*,*g* and 11*e*,*f*). Pharynx extending through 6–7 segments, tooth on anterior margin. Barrel-shaped proventricle similar in length to pharynx, extending through 5–6 segments, with 37–39 muscle cell-rows (figure 10*a*). Anal cirri with 4–6 articles (figure 11*g*).

**Remarks**. *Haplosyllis vassiae* n. sp. is similar to the Australian species *Haplosyllis imajimai* Lattig, Martin & San Martín, 2010, in the morphology of chaetae, with similar length of MJP and teeth comparable in size. However, the MF of anterior chaetae is shorter in *H. imajimai* than in the new species and they also differ in the morphology of aciculae. In addition, the alternation pattern of cirri is very different in both species, since in *H. imajimai* all cirri are similar in length [29]. Similarly, chaetae and aciculae of *H. niphatesicola* and *Haplosyllis aciculata* Lattig, Martin & Aguado, 2010 slightly resemble those in *H. vassiae* n. sp., although the MJP in these species is shorter and curved in posterior chaetae, while straight in the new species [35,38]. In addition, all chaetae in *H. niphatesicola* and *H. aciculata present* spines on the MF and the alternation pattern of anterior cirri is very different between the former and *H. vassiae* n. sp. [35,38].

**Habitat**. Symbiont of unidentified yellow sponge, 2 m depth.

**Distribution**. West Atlantic Ocean, Bermuda Archipelago.

**Etymology**. Named after the spongologist Dr Vasiliki Koutsouvelii (GEOMAR), Vassia, colleague and friend of A.V. and P.Á.-C. for her help, encouragement and friendship throughout the years.

Genus ***Opisthosyllis*** Langerhans, 1879

*Opisthosyllis* Langerhans, 1879 [29, p. 541]

**Type-species.** *Opisthosyllis brunnea* Langerhans, 1879

**Diagnosis**. Large body with numerous segments, usually with large and articulated appendages, all similar in length, except for tentacular cirri which are much longer than the rest. Pharynx with a tooth situated in the posterior end of the pharynx. Parapodia with compound chaetae, with dorsal and ventral simple chaetae usually present in posterior segments. Reproduction by squizogamic scissiparity [5].

*Opisthosyllis* sp.

Figure 12

**Figure 12.**
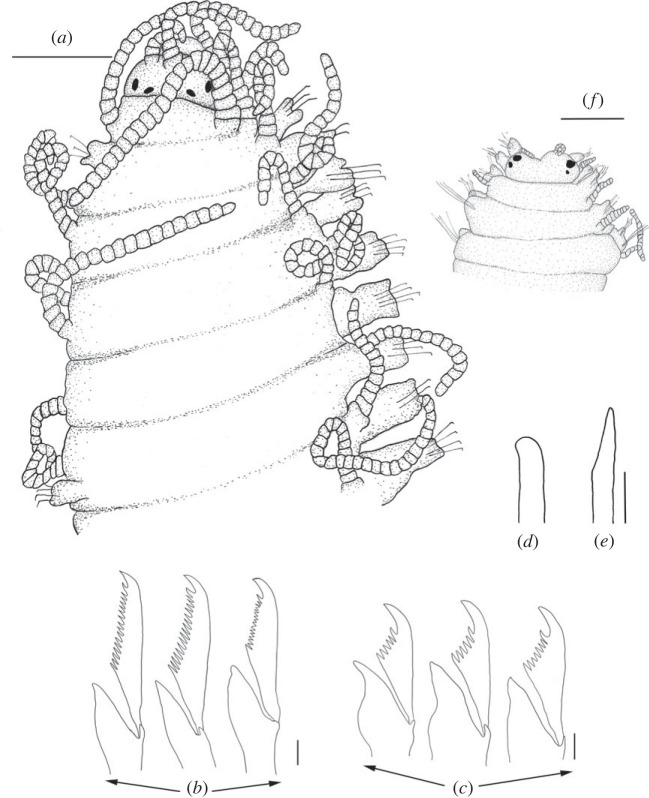
*Opisthosyllis* sp. line drawing (MNCN 16.01/19313). (*a*) Anterior part, dorsal view. (*b*) Compound chaetae, anterior parapodia. (*c*) Compound chaetae, posterior parapodia. (*d*) Acicula, anterior parapodia. (*e*) Acicula, posterior parapodia. (*f*) Tetracerous stolon. Scale bars: (*a*) 0.195 mm; (*b–e*) 5 µm; (*f*) 0.375 mm.

**Material examined**. One specimen (MNCN 16.01/19313), fixed in 96% EtOH. Bermuda, Whalebone Bay, St George's Island (32°21′53″ N, 64°42′44″ W), green algae, 2 m depth, 8 August 2014.

**Comparative material**. *Opisthosyllis brunnea* Langerhans, 1879. One specimen (MNCN 16.01/11255), Playa Antonio, Pinar del Río, Guanahacabibes Peninsula, Cuba, 21°54′09.83″ N, 84°39′34.76″ W, in intertidal rhodophyte algae, 1.8 m depth, 15 July 2006. One specimen (MNCN 16.01/11468), Uvas Islands, Gulf of Chiriquí, Panama, 7°49′00″ N, 81°46′00″ W, coral rubble, 6 m depth, 6 February 1997. One specimen (MNCN 16.01/8995), Playa Blanca, Las Palmas, Canary Islands, 28 June 1996. *Opisthosyllis viridis* Langerhans, 1879. One specimen (MNCN 16.01/11553), Uvas Islands, Gulf of Chiriquí, Panama, 7°49′00″ N, 81°46′00″ W, 6 m depth, 6 February 1997.

**Diagnosis**. Robust and thick body. All chaetae bidentate, with distal tooth much larger than proximal one, especially in posterior parapodia, where it is difficult to distinguish the proximal tooth from most distal spines on blade margin. Posterior shafts very thick, with a very prominent distal spur.

**Description**. Incomplete specimen with short and robust body, 5 mm long, 0.8–0.9 mm wide, with 33 segments (figure 12*a*). Prostomium ovate, wider than long, with two pairs of eyes in trapezoidal arrangement, with anterior eyes slightly smaller than posterior ones (figure 12*a*). Triangular palps, shorter than prostomium (figure 12*a*). Central antenna inserted in the middle of prostomium, with 22 articles (figure 12*a*). Lateral antennae on anterior margin of the prostomium, slightly shorter than central one, with 15 articles (figure 12*a*). Dorsal tentacular cirri thick and long, with 35 articles, ventral ones similar in length to antennae, with 10 articles (figure 12*a*). Dorsal cirri of all chaetigers similar in length: anterior cirri with 17–20 articles, mid-body cirri with 18–19 articles and posterior cirri with 20–22 articles (figure 12*a*). Ventral cirri shorter than parapodia. Relatively short parapodial lobes, oval shaped, longer than wide. Anterior parapodia with 7–8 compound bidentate chaetae, with distal tooth much larger than proximal one; proximal tooth slightly curved upward, similar in size to the numerous spines on the margin (figure 12*b*). Shaft of anterior chaetae with distal spines in some cases. Mid-body parapodia similar to anterior ones, but shorter and thicker. Posterior parapodia with six robust and shorter compound bidentate chaetae, with a very large distal tooth; proximal tooth difficult to distinguish from the spines of margin (figure 12*c*). Posterior shafts very thick, with a pronounced spur, especially in most ventral chaetae (figure 12*c*). Like in anterior and mid-body chaetae, posterior shaft with few distal spines, not always visible. Simple chaetae not observed. One single thin acicula in all parapodia, straight and distally blunt in anterior parapodia (figure 12*d*) and acuminate in posterior ones (figure 12*e*). Pharynx through 7–8 segments, with a minute tooth in the posterior part. Proventricle through 10 segments. Pygidium not observed.

**Figure 13.**
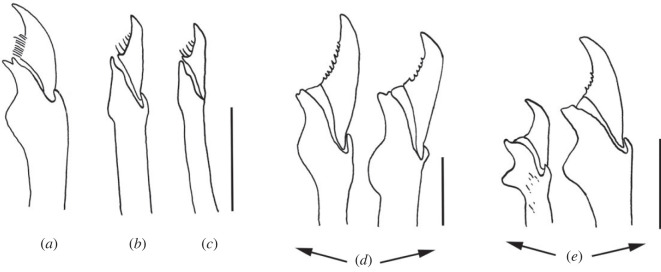
Line drawing of *Opisthosyllis brunnea* chaetae from Madeira (*a*), Japan (*b,c*) and Australia (*d,e*). (*a*) Compound chaeta. (*b*) Compound chaeta, anterior parapodia. (*c*) Compound chaeta, posterior parapodia. (*d*) Compound chaetae mid-body parapodia. (*e*) Compound chaetae, posterior parapodia. Scale bars: (*b,c*) 11.3 µm; (*d*,*e*) 40 µm. Adapted from Langerhans, 1879 (*a*), Jong, 1994 (*b,c*) and San Martín *et al.,* 2008 [39] (*d*,*e*).

**Remarks**. *Opisthosyllis* sp. resembles *Opisthosyllis brunnea* Langerhans, 1879 from Madeira in the morphology of the chaetae (figure 13*a*). This species has been reported worldwide, showing a characteristic spur in posterior shafts, although Japanese specimens have a less pronounced spur [39,40] (figure 13*b,c).* However, the new species greatly differs from Australian specimens of *O. brunnea* in the shape of the posterior since the protrusion of the shafts takes on a different shape (figure 13*d,e*). There are also marked differences in the morphology of the spines on the blades of mid-body and posterior chaetae: in the new species *Opisthosyllis* sp. they are as pronounced as in anterior chaetae, while in *O. brunnea* they are much smaller in the posterior chaetae and even absent in some cases (figures 12 and 13). On the other hand, the blades of the Madeiran [41] and Japanese [40] specimens are unidentate, while in *Opisthosyllis* sp. only the posterior ones seem to be unidentate **(**figures 12 and 13*a–c*). In general, anterior chaetae of the new species are similar to those of the revised specimens from Canary Islands, Cuba and Panama, showing the characteristic spur. However, all of them present differences in the length of blades of posterior chaetae and in the robustness of the body. The new species also resembles *Opisthosyllis viridis* Langerhans, 1879 from Madeira and *Opisthosyllis japonica* Imajima, 1966 from Japan in the morphology of its chaetae, posterior chaetae almost unidentate with a very reduced proximal tooth [39]. However, unlike *Opisthosyllis* sp., both species have simple chaetae, their appendages are thinner and longer, and present dorsal papillae [39]. It is likely that *Opisthosyllis* sp. also represents a new species; we cannot formally describe it herein given the paucity of specimens. Since only one individual is available, we could not examine it through SEM, and thus, detail descriptions of some morphological features as well as possible intraspecific differences are not provided. In any case, we consider that describing the morphology of this unknown species might be useful for future studies in the region.

**Reproduction**. Squizogamy. One female tetracerous stolon with two pairs of eyes and one pair of small unarticulated antennae (figure 12*f*).

**Habitat**. Among green algae, 2 m depth.

**Distribution**. West Atlantic Ocean, Bermuda Archipelago.

Genus ***Syllis*** Lamarck, 1818.

*Syllis* Savigny in Lamarck, 1818 [42, p. 318].

*Ioida* Johnston, 1840 [43, p. 231].

*Trichosyllis* Schmarda, 1861 [44, p. 73].

*Gnathosyllis* Schmarda, 1861: 69.

*Isosyllis* Ehlers, 1864 [45, p. 251].

*Pagenstecheria* Quatrefages, 1865 [46, p. 17].

*Aporosyllis* Quatrefages, 1865 [43, p. 87].

*Heterosyllis* Kinberg, 1866 [47, p. 248].

*Thoe* Kinberg, 1866 [44, p. 42].

*Laomedora* Kinberg, 1866 [44, p. 249].

*Eurymedusa* Kinberg, 1866 [44, p. 249].

*Chaetosyllis* Malmgren, 1867 [48, p. 162].

*Typosyllis* Langerhans, 1879 [29, p. 528].

*Ehlersia* Quatrefages, 1865 [43, p. 104].

*Langerhansia* Czerniavsky, 1881 [49, p. 395].

*Reductotyposyllis* Hartmann-Schröder, 1974 [50, p. 123].

**Type-species**. *Syllis monilaris* Savigny in Lamarck, 1818 [40, p. 317].

**Diagnosis**. Medium or large cylindrical body with numerous segments and articulated appendages. Parapodia with compound, bidentate or unidentate chaetae, sometimes with pseudospinigers. Simple chaetae on posterior parapodia and, in some cases, also on anterior ones. Reproduction by squizogamic scissiparity, with stolons of various types, depending on species [5,9].

*Syllis laiae* n. sp.

Figures 14 and 15

**Figure 14.**
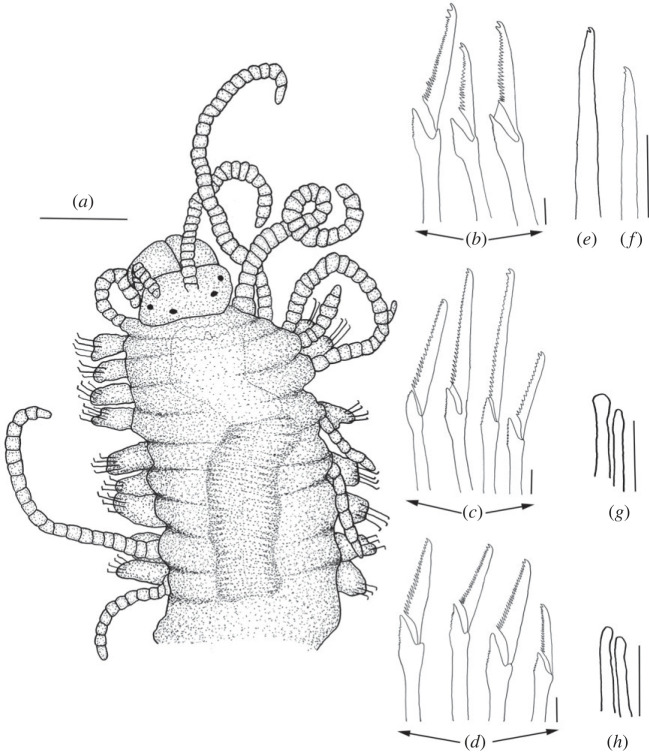
*Syllis laiae* n. sp. line drawing (MNCN 16.01/19314). (*a*) Anterior part, dorsal view. (*b*) Compound chaetae, anterior parapodia. (*c*) Compound chaetae, mid-body parapodia. (*d*) Compound chaetae, posterior parapodia. (*e*) Dorsal simple chaeta, mid-body parapodia. (*f*) Ventral simple chaeta, posterior parapodia. (*g*) Aciculae, anterior parapodia. (*h*) Aciculae, posterior parapodia. Scale bars: (*a*) 0.195 mm; (*b–d*) 5 µm; (*e*,*f*) 10 µm; (*g,h*) 5 µm.

**Figure 15.**
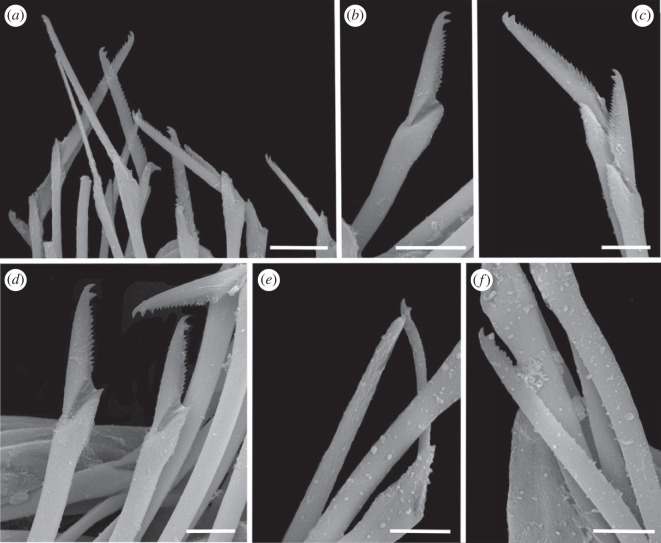
Scanning electron micrographs of *Syllis laiae* n. sp. (MNCN 16.01/19315). (*a*) Compound chaetae, anterior parapodia. (*b*) Compound chaetae, mid-body parapodia. (*c*) Dorsal compound chaetae, posterior parapodia. (*d*) Ventral compound chaetae, posterior parapodia. (*e*) Simple ventral chaetae, posterior parapodia. (*f*) Simple dorsal chaetae, posterior parapodia. Scale bars: (*a–d*) 10 µm; (*e*,*f*) 5 µm.

Zoobank ID: urn:lsid:zoobank.org:act:47438D6E-2351-41A3-9563-B68AD3A99DE7

**Material examined**. Holotype (MNCN 16.01/19314), mid-body segments of two paratypes used for molecular analyses (MNCN 16.01/19315; MNCN 16.01/19316), one mounted for SEM and the other fixed in 96% EtOH. Bermuda, Whalebone Bay, St George's Island (32°21′53″ N, 64°42′44″ W), green algae, 2 m depth, 8 August 2014.

**Comparative material**. *Syllis prolifera* Krohn, 1852. One specimen (MNCN 16.01/13286), Cala Ratjada, Mallorca, Balearic Islands, Spain, in algae, 4 m depth, 21 July 2008. One specimen (MNCN 16.01/14896), Cala Benimela, North of Menorca, Balearic Islands, Spain, in algae. One specimen (MNCN 16.01/775), Cayo Matías, Island of Pinos, Canarreos Archipelago, Cuba, in *Turbinaria turbinata*, 3 m depth. *Syllis columbretensis* (Campoy, 1982). One specimen (MNCN 16.01/7915), north of Mallorca, Balearic Islands, Spain, (39°44′33″ N, 02°33′51″ E), 72–74 m, 25 June 1994. One specimen (MNCN 16.01/7927), North of Columbrete Grande, Columbretes Islands, Castellón, Comunidad Valenciana, Spain (39°54′ 02″ N, 00°41′ 15″ E), 47 m depth, 12 July 1994. *Syllis corallicola*, Verrill, 1900. One specimen (MNCN 16.01/6874), Salamansa, St Vicent Island, Cape Verde, 20 August 1985. One specimen (MNCN 16.01/768), Punta Pedernales, Island of Pinos, Canarreos Archipelago, Cuba, 4 m depth.

**Diagnosis**. Species with a small sub-pentagonal prostomium with compound bidentate chaetae with both teeth of similar size and some spines on margins; blades decreasing in length in posterior parapodia. Dorsal and ventral simple bidentate chaetae on posterior parapodia. Two aciculae distally blunt in all parapodia.

**Description**. Holotype: complete specimen with small body, 2–3 mm long, 0.4 mm wide, with 33 segments. Small, sub-pentagonal prostomium, with four eyes in trapezoidal arrangement, anterior eyes are slightly larger than posterior ones (figure 14*a*). Palps rounded and small, smaller in length than width of prostomium. Central antenna inserted in anterior margin of prostomium, longer than lateral ones. Central antenna with 20–22 articles and lateral ones with 10–12 articles (figure 14*a*). First segment shorter than chaetigers. Dorsal tentacular cirri shorter than central antenna, with 18–19 articles. Ventral tentacular cirri with 9–10 articles (figure 14*a*). Cirri of anterior chaetigers longer than mid-body and posterior ones, with 20–25 articles; cirri of mid-body parapodia with 10–14 articles (figure 14*a*). Short and conical parapodial lobes, covering ventral cirri (figure 14*a*). Anterior parapodia with 7–9 compound chaetae, mid-body parapodia with 6–8 and posterior parapodia with 5–6 (figure 14*a*). All chaetae bidentate, both teeth similar in size, short with slightly upwardly directed spines on blade margins (figures 14*b–d* and 15*a–d*). Posterior chaetae decreasing in length (figures 13*c* and 14*d*). Shafts of all chaetae with few distal spines (figures 14*b–d* and 15*a–d*). Dorsal bidentate simple chaetae in mid-body and posterior parapodia, longer than ventral one with very small spines on margin (figures 14*e* and 15*e*). Ventral simple bidentate chaetae only in posterior parapodia, both teeth similar in size, shorter than dorsal ones with spines on margin. (Figures 14*f* and 15*f*). One or two aciculae on each parapodium, distally blunt (figure 14*g,h).* Pharynx and proventricle equal in length, both through 6–7 segments. Pharynx with pharyngeal tooth in anterior margin (figure 14*a*). Pygidium with two anal cirri with 4–5 articles.

**Remarks**. *Syllis laiae* n. sp. resembles *Syllis prolifera* Krohn, 1852 and *Syllis columbretensis* (Campoy, 1982) from the Mediterranean Sea and *Syllis compacta* Gravier, 1900 from the Red Sea in the general shape of the body and the morphology of chaetae and aciculae [5]. However, pharynx and proventricle in *S. compacta* are much longer than those in the new species extending through 13 and 12 segments respectively in the type specimens [51] and through 8 and 10 in Mediterranean individuals [52]. Anterior chaetae in *S. laiaei* n. sp. have larger blades than the ones described in *S. prolifera*, *S. columbretensis* and *S. compacta*, and posterior chaetae have longer spines on margin in these three species [5,51–53]. In addition, *S. compacta* also differs in the proximal tooth of mid-body and posterior chaetae, which is considerably smaller than distal one [52] while *S. prolifera* from Mallorca does not have simple chaetae. By contrast, *S. compacta* presents dorsal and ventral simple chaetae in mid-body and posterior chaetigers, but the morphology of the dorsal one is very different to the chaetae in *S. laiae* n. sp., since they are bidentate upwards directed and do not present spines on margin [52]. On the contrary, simple chaetae in *S. columbretensis* are very similar to the new species, but they considerably differ in the aciculae and the cirri, which are larger in this species than in *S. laiae* n. sp.

**Habitat**. Among green algae, 2 m depth.

**Distribution**. West Atlantic Ocean, Bermuda Archipelago.

**Etymology**. Species named after Laia Moreno, colleague and friend of the authors, for helping the first author with syllid identifications and for her great team spirit during her time working at P.Á.-C.'s laboratory.

### 3.2. Phylogenetic analyses

**Figure 16.**
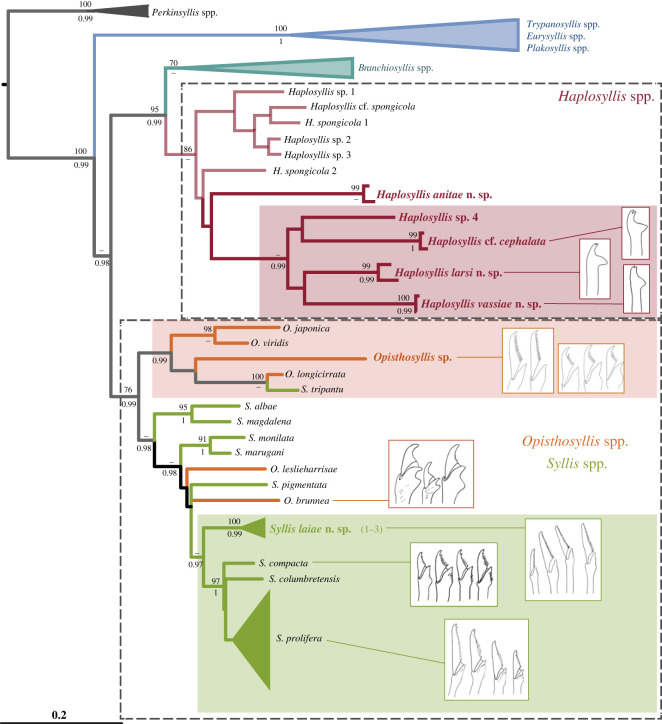
Most probable phylogenetic tree inferred from the maximum-likelihood analysis of the concatenated molecular dataset (*COI, 18S rRNA*, *16S rRNA, 28S rRNA*). Bootstrap support values (only greater than or equal to 80%) indicated above branches and posterior probability support values (only greater than or equal to 0.90) indicated below branches. The two main clades (i.e. *Haplosyllis* and *Opisthosyllis*-*Syllis*) are marked with dotted lines. Clades including Bermudan species are indicated with coloured rectangles. Insets show line drawings of posterior chaetae.

To confirm and assess the phylogenetic position of the six newly discovered species within the subfamily Syllinae and to investigate their evolutionary relationships, a final concatenated alignment comprising 3632bp (*18S* dataset, 2110bp; *16S*, 522bp; *COI*, 623bp; *28S*, 377bp) was used for phylogenetic analyses. From the 13 Bermudan specimens sequenced, one of them, *Haplosyllis* sp. 4, could not be identified due to the poor condition of the single individual collected (lacking cirri and most chaetae), but the phylogenetic analyses suggested it represents a distinct previously unknown species (figure 16; electronic supplementary material, figure S1). In addition, we were not able to amplify DNA from the new species *H. guillei* n. sp. and thus it was not included in the molecular phylogenetic analysis. The phylogenetic trees obtained with both ML and BI analyses recovered identical topologies (figure 16; electronic supplementary material, figure S1) and, therefore, the ML tree is presented with both bootstrap support (BS) and posterior probability (PP) values indicated on each node (figure 16). The genus *Haplosyllis* was recovered as a monophyletic group, but only supported in the ML analysis (figure 16; electronic supplementary material, figure S1). By contrast, the BI analysis places all *Haplosyllis* species from Bermuda except one, *H. anitae* n. sp., in a well-supported clade (figure 16; electronic supplementary material, figure S1). Noteworthy, the relationships among the different *Haplosyllis* species analysed are not well resolved in either the ML or BI results (figure 16; electronic supplementary material, figure S1). On the other hand, the genera *Syllis* and *Opisthosyllis* appear as polyphyletic groups, as a mix of species from both genera comprising a well-supported clade in both ML and BI analyses (figure 16; electronic supplementary material, figure S1). Furthermore, only the BI analysis places both new species of *Opisthosyllis* and *Syllis* in well-supported clades (figure 16; electronic supplementary material, figure S1). Thus, these results suggest that *Opisthosyllis* sp*.* is closely related to *O. japonica*, *O. viridis*, *O. longicirrata* Monro, 1939 and *S. tripantu* Álvarez-Campos & Verdes, 2017 (PP = 0.99), but not to the other morphologically similar species *O. brunnea* (figure 16; electronic supplementary material, figure S1). The new species *Syllis laiae* n. sp. is closely related to the morphologically similar species *S. compacta* and *S. prolifera* (PP = 0.97) (figure 16; electronic supplementary material, figure S1). Nevertheless, most of the relationships among the different *Opisthosyllis* and *Syllis* included in the analyses are not well resolved in either ML or BI analyses.

## 4. Discussion

The present study has expanded the knowledge on the diversity of the family Syllidae, and specifically in the subfamily Syllinae, by increasing the number of known species in the genera *Haplosyllis*, *Opisthosyllis* and *Syllis*. With the new species described herein, *Haplosyllis* contains now 42 valid species (6 in Bermuda), *Opisthosyllis* comprises 19 valid species (2 in Bermuda), and the type genus, *Syllis*, includes now 180 valid species (4 in Bermuda). We provide molecular information for all the new species, except *Haplosyllis guillei* n. sp., in addition to molecular data of *Haplosyllis* cf. *cephalata* and for an unidentified *Haplosyllis* sp. 4. Thus, *Haplosyllis* represents the Syllidae genus with most species described in Bermuda to date, and the only one with both morphological and molecular information available for all new species described in the area.

In addition, the phylogenetic analyses show that *Haplosyllis* is the only monophyletic genus, although it is only well supported in the ML analysis (figure 16; electronic supplementary material, figure S1). The inclusion of more species and specimens of this genus in a phylogenetic analysis would confirm if the taxonomical status of this group needs a careful revision, as has previously been the case with several other genera within the family Syllidae [20,54]. It is worth noting that the traditional synapomorphy of *Haplosyllis* (i.e. presence of simple bidentate chaetae) is also present in other Syllinae genera and, thus, it might result phylogenetically uninformative, given that this character has been considered an adaptation for a symbiotic way of life [13]. Our results also show that most of the Bermudan *Haplosyllis* species conform a well-supported clade in the BI analysis, but they do not share any morphological synapomorphy except for the presence of LMF shorter or equal to SW in anterior chaetae (figure 16). As we explained in detail in the taxonomic section, all the described species present several unique features, mainly related to the dorsal cirri and specific morphological features in chaetae, and thus is likely to be uninformative.

On the other hand, and in agreement with previous studies in the subfamily Syllinae [6,8,9,11] both *Opisthosyllis* and *Syllis* have been recovered as polyphyletic, since all their species cluster together in the same well-supported clade (figure 16; electronic supplementary material, figure S1). The type species of *Opisthosyllis*, *O. brunnea*, together with the Californian *O. leslieharrisae*, appear separated from the rest of the species in the genus that comprises a well-supported clade in the BI analysis, although they also cluster with a *Syllis* species (figure 16; electronic supplementary material, figure S1). In fact, this clade contains the *Opisthosyllis* sp. which is closely related to *O. japonica*, *O. viridis*, *O. longicirrata* and *S. tripantu*, all sharing a characteristic chaetal pattern, with anterior bidentate chaetae with long blades and spines on margin that are reduced in posterior chaetae, where proximal tooth also becomes almost indistinguishable from spines (figure 16; electronic supplementary material, figure S1). Thus, our results suggest that this chaetal pattern might be more phylogenetically informative than the specific details in chaetae that are traditionally used to differentiate species (e.g. presence of shafts with a pronounced spur). Nevertheless, the current molecular information available for *O. brunnea* comes from an Australian individual (electronic supplementary material, table S2), and thus, we decide not to take any taxonomic action until new fresh material can be collected from the type locality in Madeira. Given the amount of putative worldwide distributed species in the subfamily Syllinae that have resulted to be a complex of different species with restricted geographical distributions [10,20,55], any taxonomic action in this group should always be based in analyses that include material from the type locality. Finally, our phylogenetic results place the species *Syllis laiae* n. sp. within a well-supported clade in the BI analysis, closely related with the species *S. compacta* and *S. prolifera* (figure 16; electronic supplementary material, figure S1). As we reported above, some morphological similarities between the chaetae of these species can be observed, since all of them are bidentate with both teeth similar in size, they present short spines on margins, and the length of blades decreases towards the posterior end (figure 16 and taxonomic section). However, these subtle details are found in other *Syllis* species and, therefore, they cannot be considered synapomorphies of the group. Other characters, such as the morphology of reproductive stolons, have also been proposed as an important character to reorganize *Syllis* species [9,11], but, unfortunately, this feature has not been observed in our new described species. With the current lack of ecological or developmental information for most *Syllis* species, and the fact that traditional diagnostic characters are evolutionarily labile and may not accurately reflect evolutionary histories, it would be worth considering to reorganize this complicated genus according to molecular characters, as it has been proposed in other marine invertebrates, including annelids [56–58].

## 5. Conclusion

In the present study, we have increased the number of known species of Syllidae in the Bermuda Archipelago with the description of five new species from the genera *Haplosyllis* and *Syllis*. Specifically, we have considerably expanded the current morphological and molecular information available for *Haplosyllis* species, which seem to be the most abundant genus in this region. We have also shown that both *Syllis* and *Opisthosyllis* genera are not monophyletic, while *Haplosyllis* genus was recovered as a monophyletic group only in one of the phylogenetic analyses. In addition, the relationships among the different species analysed are not well resolved for any of the three genera. Therefore, we emphasize the necessary work to improve the phylogenetic inference in Syllinae genera and to establish the connections between phenotype and genotype that have generated the huge morphological variability observed in the group.

## Data Availability

Specimens used were deposited at the National Museum of Natural Sciences of Madrid (MNCN-CSIC). Newly generated DNA sequences were deposited in NCBI Genbank under the accession numbers: for *28S rRNA* (OQ947172–OQ947177); *18S rRNA* (OQ947189–OQ947194); *COI* (OQ947289–OQ947194) and *16S rRNA* (OQ946604–OQ946607). The new species described have been registered in ZooBank: *Haplosyllis anitae* n. sp. (Zoobank ID: urn:lsid:zoobank.org:act:34A34EA8-60C3-4CEF-BCE8-525F7F330B44); *Haplosyllis guillei* n. sp. (Zoobank ID: urn:lsid:zoobank.org:act:D7E908A5-8B41-469B-A1BB-B0CA510E4C5D); *Haplosyllis larsi* n. sp. (Zoobank ID: urn:lsid:zoobank.org:act:E7C2D753-E388-4562-BD1C-0824871305A9); *Haplosyllis vassiae* n. sp. (Zoobank ID: urn:lsid:zoobank.org:act:A036DDE1-1156-45BE-94D0-096A45CF72B2); *Opisthosyllis* sp. and *Syllis laiae* n. sp. (Zoobank ID: urn:lsid:zoobank.org:act:47438D6E-2351-41A3-9563-B68AD3A99DE7). The data are provided in electronic supplementary material [59].
